# Digital Intervention With Lifestyle Coach Support to Target Dietary and Physical Activity Behaviors of Adults With Nonalcoholic Fatty Liver Disease: Systematic Development Process of VITALISE Using Intervention Mapping

**DOI:** 10.2196/20491

**Published:** 2021-01-15

**Authors:** Kate Hallsworth, Stuart McPherson, Quentin M Anstee, Darren Flynn, Laura Haigh, Leah Avery

**Affiliations:** 1 Liver Unit Newcastle upon Tyne Hospitals NHS Foundation Trust Newcastle upon Tyne United Kingdom; 2 Newcastle NIHR Biomedical Research Centre Newcastle upon Tyne United Kingdom; 3 Translational and Clinical Research Institute Faculty of Medical Sciences Newcastle University Newcastle upon Tyne United Kingdom; 4 Centre for Rehabilitation School of Health & Life Sciences Teesside University Middlesbrough United Kingdom

**Keywords:** nonalcoholic fatty liver disease, internet-based intervention, lifestyle, diet, physical activity, weight loss

## Abstract

**Background:**

Nonalcoholic fatty liver disease (NAFLD) is linked to excessive calorie consumption, physical inactivity, and being overweight. Patients with NAFLD can halt or decelerate progression and potentially reverse their condition by changing their lifestyle behavior. International guidelines recommend the use of lifestyle interventions; however, there remains a discordance between published guidelines and clinical practice. This is primarily due to a lack of NAFLD-specific interventions to support weight loss and improve liver function.

**Objective:**

This study aims to use intervention mapping to systematically develop a digital intervention to support patients with NAFLD to initiate and maintain changes in their dietary and physical activity behavior to promote weight loss.

**Methods:**

Intervention mapping consisted of 6 steps: step 1 involved a needs assessment with primary and secondary health care professionals (HCPs) and patients with NAFLD; step 2 involved identification of the social cognitive determinants of change and behavioral outcomes of the intervention; step 3 involved linking social cognitive determinants of behavioral outcomes with behavior change techniques to effectively target dietary and physical activity behavior; step 4 involved the development of a prototype digital intervention that integrated the strategies from step 3, and the information content was identified as important for improving knowledge and skills from steps 1 and 2; step 5 involved the development of an implementation plan with a digital provider of lifestyle behavior change programs to patients with NAFLD using their delivery platform and lifestyle coaches; and step 6 involved piloting the digital intervention with patients to obtain data on access, usability, and content.

**Results:**

A digital intervention was developed, consisting of 8 modules; self-regulatory tools; and provision of telephone support by trained lifestyle coaches to help facilitate behavioral intention, enactment, and maintenance. A commercial provider of digital lifestyle behavior change programs enrolled 16 patients with NAFLD to the prototype intervention for 12 consecutive weeks. A total of 11 of the 16 participants successfully accessed the intervention and continued to engage with the content following initial log-in (on average 4 times over the piloting period). The most frequently accessed modules were *welcome to the program*, *understanding NAFLD*, and *food and NAFLD*. Goal setting and self-monitoring tools were accessed on 22 occasions (4 times per tool on average). A total of 3 out of 11 participants requested access to a lifestyle coach.

**Conclusions:**

Intervention mapping provided a systematic methodological framework to guide a theory- and evidence-informed co-design intervention development process for patients and HCPs. The digital intervention with remote support by a lifestyle coach was acceptable to patients with NAFLD and feasible to deliver. Issues with initial access, optimization of information content, and promoting the value of remote lifestyle coach support require further development ahead of future research to establish intervention effectiveness.

## Introduction

### Background

Nonalcoholic fatty liver disease (NAFLD) affects up to 33% of adults in western countries and is the most common liver condition worldwide [1]. Approximately 40% of patients with NAFLD will develop progressive liver fibrosis, and ultimately, 5% to 11% of patients with NAFLD will develop end-stage liver disease [2,3]. Weight loss, achieved through changes in dietary and physical activity behaviors, is the recommended treatment for NAFLD, which can reduce liver fat, inflammation, and fibrosis [4,5]. Evidence-based clinical guidelines for the management of NAFLD state the importance of lifestyle behavior change in patients with NAFLD, regardless of disease severity [6-8]. However, patients with NAFLD typically have their condition monitored rather than actively managed with minimal support to change their lifestyle behaviors [9]. A potential explanation for this evidence-practice gap is the lack of NAFLD-specific, theory- and evidence-informed interventions for use in routine clinical practice.

### Intervention Development

Intervention mapping provides a robust method for systematically developing theory- and evidence-informed interventions that integrates the perspectives and needs of the target populations [10]. It consists of 6 consecutive steps to provide a transparent account of the translation of theory- and evidence-based behavior change techniques (BCTs) into intervention components, which explicitly target theoretical determinants of behavior and behavior change [10]. This process enables replication in terms of development and delivery of interventions and facilitates intervention optimization and robust evaluation.

In the context of this study, the target populations of interest are patients with NAFLD and health care professionals (HCPs) responsible for their care. Our previous research indicated that lifestyle behavior change interventions need to be capable of meeting the needs of both patients and HCPs. This increases the likelihood that HCPs will use or refer patients to the intervention, that uptake by patients is optimal, and that the intervention is capable of successfully improving outcomes for patients with NAFLD in the long term [9-11].

We present a detailed overview of the systematic development of an NAFLD-specific digital intervention using intervention mapping [10]—VITALISE (Intervention to Promote Lifestyle Change in Nonalcoholic Fatty Liver Disease). We describe the co-design process with patients and HCPs used to develop the form and information content of VITALISE to target dietary and physical activity behaviors to support weight loss and weight loss maintenance. This enhances the transparency and replicability of the intervention, facilitates optimization post piloting, and increases the likelihood of uptake and sustainability of the intervention over time.

## Methods

### Overview of Development Process

**Figure 1:**
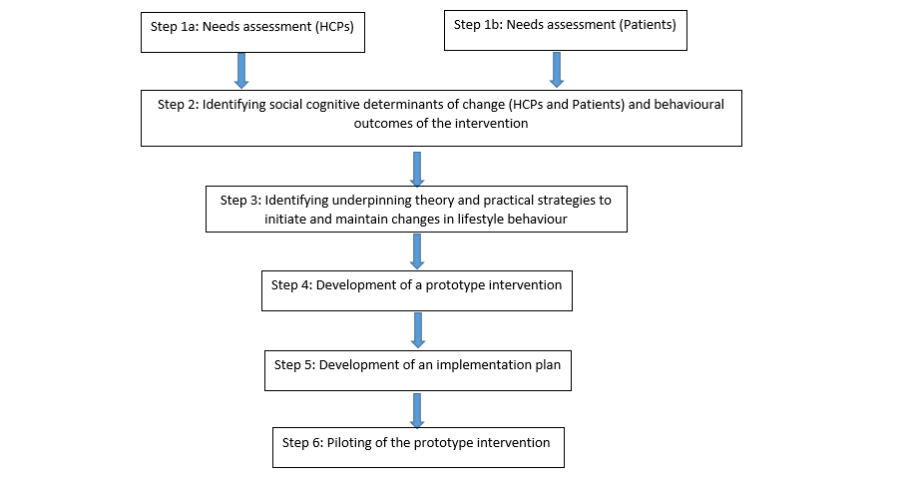
Overview of the intervention development process undertaken with reference to intervention mapping. HCP: health care professional.

A diagrammatic summary of the co-design process that corresponds to the 6 stages of intervention mapping [10] is shown in Figure 1. Ethical approval for all stages was secured from the National Health Service (NHS) London-Riverside Research Ethics Committee (reference: 15/LO/0815).

### Step 1: Needs Assessment

Step 1 of intervention mapping involved a needs assessment of the target populations (ie, HCPs responsible for diagnosing and managing patients with NAFLD and patients diagnosed with NAFLD). We referred to national and international guidelines for the diagnosis and management of NAFLD [6-8] to inform the development of topic guides for semistructured one-to-one interviews with 21 HCPs and 12 patients. Interviews were conducted either by a senior physiotherapist with expertise in NAFLD or a health psychologist with expertise in health behavior change. Both thematically analyzed the data generated. The methods and findings of these interviews have been published previously [9].

### Step 2: Defining the Objectives and Behaviors to Change (HCPs and Patients)

Step 2 involved exploring the social cognitive determinants (eg, attitudes, risk perceptions, and self-efficacy) of behavioral intention and behavior change (performance objectives) identified from the interviews with HCPs and patients during step 1. This included defining the behavioral outcomes of the intervention (change objectives, eg, dietary and physical activity behavior). Specifically, the change objectives describe how the social cognitive determinants of behavioral intention and behavior can be targeted to inform selection of practical strategies that should be used to target these determinants in step 3.

### Step 3: Identifying Underpinning Theory and Practical Strategies to Initiate and Maintain Changes in Lifestyle Behavior

Step 3 involved linking social cognitive determinants of intention and behavior (eg, attitudes, beliefs, and risk perception) with the behavioral outcomes (informed by steps 1 and 2) and selecting appropriate theory- and evidence-based intervention strategies [12] to target the behaviors of interest (ie, diet and physical activity). Selection of specific BCTs was informed by the findings of our qualitative research with HCPs and patients and involved identification of specific theoretical domains using the Theoretical Domains Framework (TDF) [11-13]. Domains identified from the perspectives of HCPs (eg, professional role and identity, skills, and knowledge) and patients (eg, knowledge, skills, and self-regulation) informed the selection of BCTs during team discussions using a valid and reliable taxonomy [12]. For example, the theoretical domain *knowledge* led to the selection of the BCTs’ *information about health consequences* and *information about antecedents* and the theoretical domain *professional role and identity* led to the inclusion of lifestyle coach support. Mapping the intervention strategies in this way enabled identification of what is likely to work and not work and serves as a mechanism to optimize the content of the intervention.

### Step 4: Development of a Prototype Intervention

In collaboration with a design team at Changing Health Limited [14], step 4 involved the development of a prototype digital intervention that integrated the theory-based practical strategies selected during step 3 to target dietary and physical activity behavior of patients to promote weight loss. A draft paper-based overview of VITALISE was developed by the research team to effectively communicate the content to the designers, which was subsequently developed into a digital draft of the intervention.

HCPs and patients with NAFLD were further engaged separately in interactive group workshops (consisting of a demonstration of the draft digital intervention, small group work, and plenary discussion) to elicit their views and preferences on the form and information content to develop a prototype intervention and to identify any issues related to acceptability and usability.

A total of 4 workshops were conducted: two with primary HCPs (group 1 involved 4 participants and group 2 involved 5 participants), one with secondary and tertiary HCPs (involving 5 participants), and one with patients (involving 9 participants). Critically, we explored the optimal way for HCPs to describe the intervention to patients in terms of its aims, objectives, content, and delivery. In addition, we sought the views of HCPs on their support requirements to follow up patients and provide feedback. Workshops were facilitated by a senior physiotherapist and health psychologist and lasted 90 min with patients and 45 min with HCPs. Detailed notes were taken throughout the interactive group sessions.

In addition, participating HCPs were given full access to the draft digital intervention to review individually and were asked to provide feedback on the content with reference to NAFLD clinical guidelines. The outcomes of the workshops and feedback from HCPs informed refinements to the draft version of the intervention to produce an optimized version of the prototype ready for piloting.

### Participants

HCPs and patients from 2 NHS hospital trusts and 11 NHS clinical commissioning groups in England were invited to take part in the study (steps 1 to 4 of the intervention mapping process). HCPs working in both hospital and community settings were purposively sampled from specialties that manage patients with NAFLD on a regular basis (including hepatology, gastroenterology, diabetology, and general practice) to ensure representation across specialties and inclusion of different professions (eg, physicians, nursing, and dietitians).

A convenience sample of patients aged ≥18 years with a confirmed diagnosis of NAFLD, regardless of disease severity, was identified by clinicians from primary, secondary or tertiary care settings. They were invited to take part in the study (steps 1 to 4 of the intervention mapping process) by a letter of invitation and asked to contact a member of the research team if they were interested in taking part.

### Step 5: Development of an Implementation Plan

Changing Health Limited [14] is a private digital lifestyle behavior change organization that has a proven track record of delivering lifestyle behavior change programs, with lifestyle coach support at scale to the NHS. They agreed to host VITALISE alongside their existing programs for type 2 diabetes management and prevention. Meetings were held with Changing Health’s digital development team and operations manager to plan and agree how patients would be given access to the intervention and who would provide technical support throughout the pilot testing described in step 6. In collaboration with Changing Health Limited, an implementation plan was developed to deliver VITALISE to patients with NAFLD using their delivery model (ie, digital intervention with remote support by lifestyle behavior coaches). The coaches provided by Changing Health were trained by a senior physiotherapist with specific expertise in NAFLD and a chartered health psychologist with expertise in lifestyle behavior change. The training was delivered face-to-face during one session lasting 2 hours.

### Step 6: Piloting the Prototype Intervention

Step 6 of the intervention mapping process involved pilot testing VITALISE with a small volunteer sample of 16 participants recruited from LiverNorth, a national liver patient support group. Individuals were eligible to participate if they had a confirmed diagnosis of NAFLD or had been advised by an HCP that they were at risk of developing the condition due to being overweight or obese. The aim was to obtain data on access and engagement with the intervention and to obtain feedback from patients to inform optimization ahead of a larger scale evaluation. Automatically logged data on access and engagement with VITALISE were analyzed using appropriate summary statistics, with qualitative data from the feedback questionnaire subjected to iterative conceptual content analysis by the author (KH). A priori and emergent coding were used to summarize key themes for discussion with the research team, who served as a challenge forum on the integrity of the analysis.

## Results

### Overview of Sample

We engaged HCPs and patients in a co-design process to develop a theory-informed digital intervention—VITALISE. Details of the participants are shown in Table 1.

**Table 1:** Summary of demographics for health care professionals and patients who participated throughout the intervention development process.

Characteristics			Values
**Health care professionals (n=21), n (%)**			
	**Gender**		
		Male	10 (48)
		Female	11 (52)
	**Specialty**		
		Hepatology (3×consultants; 1×specialist registrar)	4 (19)
		Gastroenterology (4×consultants)	4 (19)
		Diabetology (3×consultants)	3 (13)
		Primary care physician	6 (29)
		Dietician (1×weight management lead 1×diabetes lead)	2 (10)
		Hepatology specialist nurse	1 (5)
		Primary care practice nurse	1 (5)
**Patients with NAFLD^a^ (n=28)**			
	**Gender, n (%)**		
		Male	12 (43)
		Female	16 (57)
	**Characteristics**		
		Age (years), mean (SD; range)	59 (8; 42-72)
		Ethnicity (White or White British), n (%)	28 (100)
		Diagnosed NAFLD, n (%)	22 (79)
		High risk of NAFLD due to overweight or obesity, n (%)	4 (14)
		Other liver disease, n (%)	2 (7)

^a^NAFLD: nonalcoholic fatty liver disease.

### Intervention Mapping Step 1: Needs Assessment With HCPs and Patients

The findings of the interviews with 21 HCPs and 12 patients have been published previously [9], and a summary of key findings is presented here.

HCPs included hepatology (3×consultants and 1×specialist registrar), gastroenterology (4 consultants), diabetology (3x consultants), primary care physicians, dieticians (1×weight management lead and 1×diabetes lead), a hepatology specialist nurse, and a primary care practice nurse. HCPs reported a lack of knowledge, skills, and available resources to effectively target lifestyle behavior changes in patients following the diagnosis of NAFLD. Several HCPs acknowledged that targeting lifestyle behavior change should be part of routine practice. However, due to time constraints and competing clinical demands, they reported advising patients to lose weight and subsequently monitored their patients’ weight thereafter with no further lifestyle intervention. Nonspecialist HCPs reported a lack of knowledge about NAFLD and its management and emphasized the need for training to provide evidence-based information and advice to patients during consultations [9].

The lifestyle behavior change literature on NAFLD places an emphasis on a 7% to 10% weight loss target for patients to reduce their liver fat and inflammation sufficiently to improve liver health [4,5]. An incremental weight loss percentage increase is associated with better outcomes [15,16]. However, most patients interviewed reported being unaware of the association between weight loss and improvements in liver health. Importantly, none of the patients interviewed reported being aware that NAFLD was progressive and that lifestyle behavior changes could prevent or decelerate the progression of NAFLD to severe liver disease, and in some cases reverse the condition. When asked about their information needs at the time of diagnosis (ie, what would increase their intention to make lifestyle behavioral changes), they reported the need for information on risks associated with NAFLD and how NAFLD can be managed with dietary and physical activity behavior changes. The majority of patients reported being advised to lose weight by HCPs; however, this advice was regularly received because many patients had comorbid conditions, including obesity and diabetes. Therefore, they did not universally link weight loss advice to NAFLD, its development, or progression. None of the patients interviewed reported being supported to make behavioral changes. However, the majority emphasized that increased awareness that NAFLD could be effectively managed in this way, including guidance on what kinds of changes to make, how to make changes, and the required magnitude of changes, would strengthen their behavioral intentions for increasing physical activity and making dietary changes to reduce their energy consumption and composition of diet. The value of support from a trusted HCP was also emphasized. In this regard, information, feedback on progress, and social support were identified as important for a proportion of those interviewed; however, the evidence most strongly supported the need for information about NAFLD, risk of progression, and information about effective dietary and physical activity approaches [9]. Data were further analyzed using the TDF to identify specific targets for intervention. The findings have been published previously [11]. There is evidence to support a variety of dietary approaches to facilitate weight loss in the context of NAFLD (eg, Mediterranean diet [with a focus on increased fish, fruit, and vegetable intake] to promote weight loss and improve liver health, intermittent fasting, and calorie restriction to reduce energy intake); however, barriers exist to uptake and adherence. Therefore, to support individual preferences and to increase the likelihood of continued engagement, the intervention provided information on all 3 of these dietary approaches as well as links to trusted websites for more detailed information (eg, recipe ideas). As such, participants were encouraged to select the dietary approach that they felt would work best for them. This could involve reducing the amount of food they usually eat (eg, reduce portion sizes) or substitute food items usually consumed with alternatives (eg, substitute whole milk for semiskimmed or red meat for fish). In terms of physical activity and exercise, there were no specific recommendations regarding the type and amount of physical activity. Instead, the role of physical activity and exercise in the context of NAFLD was provided (eg, promotion of weight loss maintenance and reduction in liver fat) with additional information about different types of physical activities and exercises to promote an informed- and preference-based choice. The focus was on increasing everyday levels of activity.

### Intervention Mapping Step 2: Identifying Determinants of Intention and Behavior of HCPs and Patients

A total of 8 theoretical domains (out of 14) were identified from the analyses of interviews with HCPs in step 1. These were beliefs about consequences; professional role and identity; environmental context and resources; knowledge; skills; goals; behavioral regulation; and memory, attention and decision processes [11]. Although HCPs highlighted a lack of knowledge and skills to effectively target lifestyle behavior changes in their patients, the issue of competing demands (eg, clinical goals) and lack of time emerged as salient barriers. Furthermore, a small number of HCP specialists queried whether delivery of lifestyle behavior change should be part of their role (professional role and identity), that is, the requirement to deliver interventions would potentially take them away from their core clinical role. This suggested that any intervention developed for delivery by specialists may face implementation challenges. As such, we defined the target behaviors of HCPs as promotion of lifestyle behavior change (ie, emphasizing the importance in the context of NAFLD), providing advice, referring to another professional for lifestyle behavior change support (eg, to an external provider), and providing feedback on progress in relation to the impact of behavioral changes on clinical outcomes. Although it was acknowledged that there is a need for training to achieve this (eg, knowledge of what NAFLD is, how it develops, and how lifestyle behavioral change plays a vital role in the management of the disease), this perceived burden was considered less than delivering an intervention (as opposed to referring the patient) within routine consultations. HCPs perceived their patients as resistant or reluctant to change lifestyle behaviors (beliefs about consequences); however, this was largely attributable to patients reporting a lack of awareness of the links between lifestyle changes, weight, and liver health in the context of NAFLD, which inhibited them from developing an intention to change their diet and physical activity levels.

A total of 4 theoretical domains were identified from interviews with patients in step 1 as the focus for intervention. These were knowledge, beliefs about consequences, social influences, and behavioral regulation [11]. Patients emphasized a need for information, support, and feedback on progress from a trusted HCP; therefore, the performance objectives of HCPs were extended to monitor outcomes of behavioral changes with their patients to provide them with tailored feedback (ie, feedback on the impact that lifestyle behavioral changes had made on their weight status and liver health) and support.

Therefore, the objective of the patient intervention was to communicate information to target attitudes, beliefs, and risk perceptions to increase the strength of behavioral intentions. Support with planning and monitoring dietary and physical activity changes to initiate and maintain weight loss was considered important once patients were aware of the benefits of lifestyle change. Monitoring progress in particular (behavioral regulation) was identified by patients as a process that would promote self-efficacy and further support motivation.

### Intervention Mapping Step 3: Selecting Theory and BCTs

The findings of steps 1 and 2 were used to guide the selection of theory and theory-linked BCTs to structure and populate the content of the digital intervention. It became apparent upon completion of our qualitative interview study that patients were poorly informed about NAFLD, what it is, how it manifests, associated risks, and how it can be managed. Furthermore, many patients interviewed reported being advised that NAFLD was nothing to worry about. They expressed a desire to know more about NAFLD, specifically the risks of progression following their invitation to the study, that is, for many patients, it only become known by taking part in the study that NAFLD could progress to cirrhosis, for example. Only a minority of participants reported the need for support to make and sustain behavioral changes. Given the need to focus on knowledge, beliefs, and risk perception to promote intention formation, and the importance of support to achieve and maintain weight loss for a proportion of individuals interviewed, the health action process approach (HAPA) was considered an appropriate theory to underpin the development of the intervention [17].

As described in step 2, risk perception was specifically emphasized by patients as key to behavioral intention formation. Once they were aware of the potential benefits of making lifestyle behavior changes to achieve weight loss and the positive impact of this on liver health, they reported a need for support to make and sustain lifestyle behavior changes to lose weight and maintain weight loss. Table 2 provides an overview of the theoretical constructs of the HAPA, BCTs used to operationalize each construct, and a description of the mechanism underpinning each BCT.

It was important that all constructs of the HAPA were targeted with conceptually appropriate intervention components and theory-linked BCTs to fully operationalize the theory. This ensures that the intervention is replicable and can be adequately tested and increases the likelihood that the intervention will be effective for changing behaviors [18].

**Table 2:** Overview of the theoretical constructs, behavior change techniques used to operationalize each construct, and a description of their use within the digital intervention to promote lifestyle change in nonalcoholic fatty liver disease.

Theoretical construct	BCTs^a^ to operationalize constructs [12] (corresponding taxonomy code numbers)	Description and purpose of BCT use	Source of BCT selection from TDF^b^ analyses in Step 1^c^
Risk perception	Information on antecedents (4.2) Information about health consequences (5.1)	Communicate and challenge perceptions about NAFLD^d^ risk	Domain Knowledge
Outcome expectations	Pros and cons (9.2) Credible source (9.1)	Present pros and cons for making lifestyle behavior changes in relation to risk and management of NAFLD. An NAFLD specialist communicates this message to increase credibility	Domains Beliefs About Consequences and Knowledge
Planning (action and coping)	Goal setting behavior (1.1) Social support (unspecified; 3.1) Social support (practical; 3.2) Action planning (1.4) Problem solving (1.2) Instruction on how to perform a behavior (4.1) Behavioral substitution (8.2) Self-monitoring of behavior (2.3) Self-monitoring of behavior on outcomes of behavior (2.4) Review behavior goals (1.5)	Prompt and support the development of behavioral goals and plans. Problem-solving strategies, social support, self-monitoring, and feedback promote maintenance. Review goals to enhance motivation and promote maintenance	Domains Goals, Social Influences, Knowledge, and Behavioral Regulation
Self-efficacy (task and coping, recovery)	Social support (unspecified; 3.1) Social support (practical; 3.2) Self-monitoring of behavior (2.3) Self-monitoring of behavior on outcomes of behavior (2.4) Feedback on outcomes of behavior (2.7)	Provide mechanisms for ensuring risk is adequately understood, planning is realistic and within capabilities, and problem solving is explicitly linked to target behaviors (diet and physical activity). Feedback and self-monitoring to provide positive reinforcement	Domains Social Influences and Behavioral Regulation

^a^BCT: behavior change technique.

^b^TDF: Theoretical Domains Framework.

^c^Source of BCT selection from TDF analyses were identified from the analyses of interviews with HCPs and patients in step 1.

^d^NAFLD: nonalcoholic fatty liver disease.

### Intervention Mapping Step 4: Designing the Prototype Intervention

Following discussions within the research team regarding the findings in steps 1 to 3, a consensus was reached that a digital intervention would be the optimal mode of delivery for the proposed lifestyle intervention. This would facilitate standardization of the intervention delivery across primary, secondary, and tertiary care settings. Digital delivery with remote support also offers a scalable solution that can be tailored to individual patient needs. Evidence has shown that digital interventions can effectively target social cognitive determinants of behavior [19,20], which is vitally important in the context of NAFLD management with reference to our qualitative findings.

Patients highlighted the need for personalized feedback on behavioral changes (diet and physical activity) and outcomes of behavioral changes (eg, weight and liver health); therefore, the decision to include lifestyle coaches to promote continued engagement through feedback provision (specifically feedback on behaviors) and support to plan, overcome barriers, and monitor behavior was considered important. As such, alongside digital delivery, remotely delivered (telephone) lifestyle coach support was provided to maximize engagement with the intervention and to provide the desired feedback and support required by patients. Lifestyle coach support has been demonstrated to increase engagement with digital interventions [21] and the use of self-regulation strategies to target dietary and physical activity behavior within the context of type 2 diabetes prevention and management [22], which were considered transferable in the context of NAFLD. In addition, research has reported on the benefits of remotely delivered behavior change interventions [23,24], including computer-delivered interventions for effectively targeting social cognitive determinants of behavior, including risk [19].

The content of VITALISE was informed by steps 1 to 3 and was designed to increase knowledge about NAFLD, to raise awareness about the risk of NAFLD progression, and to highlight the associations with overweight or obesity to increase motivation or intention to make behavioral changes to promote weight loss. It was clear from the needs assessment that clinical team members were unable to provide this type and level of support due to training needs and competing clinical demands. The intervention was therefore designed to complement the existing care pathway and provided a means for HCPs to refer patients for lifestyle behavior change support.

**Figure 2:**
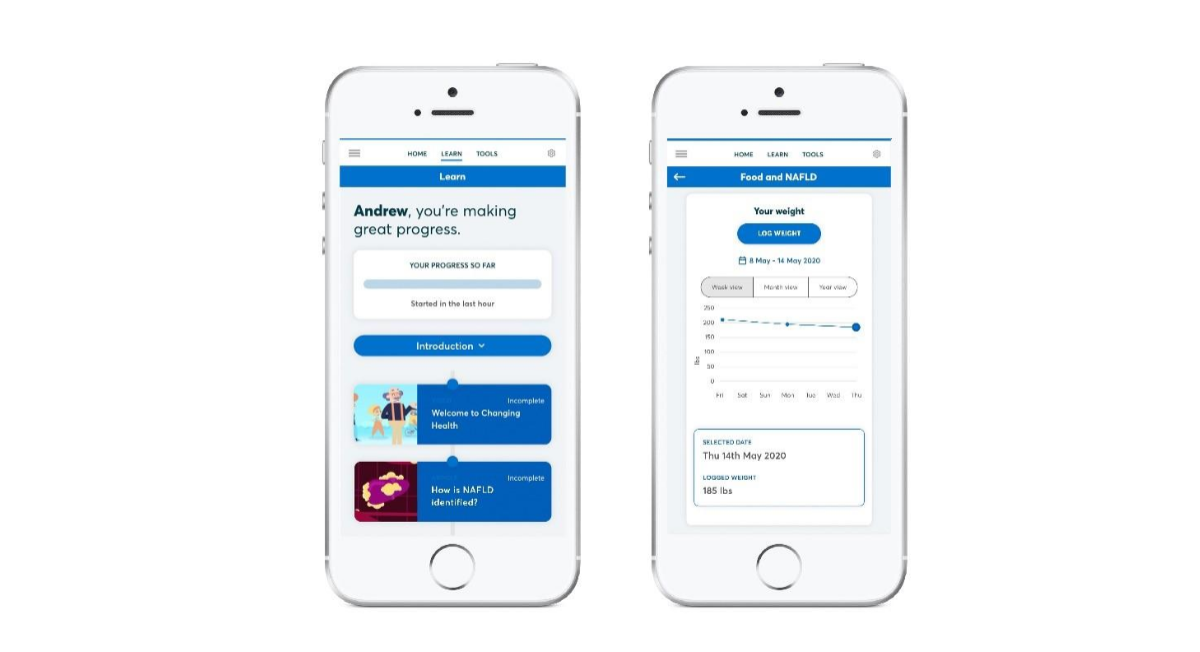
Images of the digital interVention to promote lIfesTyle change in nonAlcoholic fatty LIver diseaSE (VITALISE).

A prototype intervention was developed in collaboration with the design team at Changing Health Limited [14] and comprised 8 modules (Table 3) accessible via a home screen of the digital program (Figure 2). Participants were tunneled through the modules in a sequential order to maximize engagement with the intervention content.

Module 1 provides a message from a specialist in lifestyle management in NAFLD welcoming patients to the program and presents a summary of the aims and objectives. Module 2 presents information on NAFLD (how it is identified or diagnosed, risk of progression, understanding test results, management strategies, and the potential long-term complications of NAFLD when left unmanaged) and patient narratives reporting on success stories. Module 3 provides information on how to access a lifestyle coach, how to make the most of coaching, and how to book follow-up sessions. Module 4 is specifically about food in the context of NAFLD and describes the role of macronutrients, nutrition, and energy balance. Module 5 uses the fundamentals of module 4 to provide information on selecting an appropriate dietary approach (eg, Mediterranean diet [25] and calorie restriction) to initiate and maintain weight loss and improve liver health. Module 6 covers understanding food labels and portion sizes as well as practical strategies to overcome barriers to maintaining dietary behavior change. Module 7 focuses on the role of physical activity and exercise in the context of NAFLD, weight loss, and weight loss maintenance, with an emphasis on the type and amount of physical activity or exercise required to derive benefits on liver health. Finally, module 8 presents a suite of tools to facilitate self-regulation (eg, behavioral goal setting, feedback and self-monitoring tools for diet, physical activity, and weight). As a whole, the content of VITALISE incorporates recommendations made in national and international clinical guidelines for the management of patients with NAFLD [7,8,26].

Once enrolled in the study, patients received a welcome email with a unique link to access VITALISE using their personal email address. The information provided in each module is presented in a range of formats (eg, written text articles, interactive tools, and animations) to engage users and support encoding of information. Upon logging in, patients are asked to enter their height, weight, and weight loss goal. This information populates the self-regulation tools to facilitate tracking of progress and to generate automated feedback to support self-efficacy. Patients can upload details of their food choices (using photographs or a written description), daily step-count, and weight to enable self-monitoring.

Once participants had completed all 8 digital modules in a sequential order, they were granted access to a lifestyle coach. No specific recommendations on the frequency of contact with the coach were specified, although it was restricted to an initial 20 min appointment, followed by weekly 10 min appointments over the 12-week intervention period. The coaching sessions focused on details of participants’ dietary choices, daily step counts or time spent active, and weight status to facilitate personalized goal setting and problem solving, with subsequent sessions focusing on review of behavioral goals and feedback on behavior.

Feedback on the prototype intervention from HCPs and patients in separate workshops provided evidence that the interactive components were useful and provided acceptable and accessible representations of what otherwise would have been complex information to communicate (eg, NAFLD progression). Feedback from both groups facilitated minor modifications to the written content of the intervention, including a clearer description of the noninvasive measures used to help identify disease severity in NAFLD, and the addition of links to healthy recipe ideas to support dietary behavior change.

**Table 3:** Overview of module content.

Modules and subcomponents		Description of information content (BCT^a^ code numbers)
**Module 1: Introduction**		
	Welcome to the program	Message from a specialist in lifestyle management of NAFLD^b^ providing an overview of the aims and objectives of the program (9.1)
**Module 2: Understanding NAFLD**		
	What is NAFLD?	Overview of the liver, what is NAFLD and how it progresses, and the different stages of the disease (4.2, 5.1)
	How is NAFLD identified and diagnosed?	Overview of the tests and investigations that may be used to diagnose NAFLD (5.1, 5.2)
	Understanding test results	What the different tests and investigations measure and what the results mean (5.1)
	NAFLD myths	Common myths about NAFLD and why it should not be left unmanaged (4.2)
	Managing your NAFLD	Animations demonstrating how lifestyle change can improve liver health and reduce cardiovascular disease risk and improve metabolic control (5.1, 5.2)
	Patient success stories	Real-life examples of how people with NAFLD or at risk of NAFLD have improved their liver health by making lifestyle changes (6.2)
	Long-term complications of NAFLD	Potential consequences of not managing NAFLD (5.1, 9.2)
**Module 3: Getting started with coaching**		How to access lifestyle coaches and what to expect from coaching sessions (4.1)
**Module 4: Food and NAFLD**		
	Energy balance	How to balance energy requirements in the context of weight loss (2.7, 4.1, 8.2)
	Nutrition and NAFLD	The relationship between diet quality, eating patterns, and NAFLD (4.2, 5.1, 5.2)
	Understanding carbohydrates	What are carbohydrates and the role they play in diet (5.1)
	Understanding fats	What are fats and the role they play in our diet (5.1)
	Understanding alcohol	Recommended alcohol intake, calorie content in alcoholic drinks, and provision of a web-based alcohol calculator (2.3, 5.1)
**Module 5: How do I make changes to my diet?**		
	Finding the right dietary approach	Animation explaining the evidence supporting 2 dietary approaches for managing NAFLD (5.2, 9.1)
	Calorie restriction	Calorie restriction in more detail, provision of web-based weight loss calculators and calorie checkers, and link to the NHS^c^ weight loss plan (2.7, 4.1, 5.1)
	Mediterranean diet	Mediterranean diet in more detail (5.1)
	Alternative dietary approaches	Information and external links to alternative dietary approaches (9.1)
**Module 6: Practical tips**		
	Supermarket tour	Animation virtually walking the user around a supermarket providing practical tips on what to look out for when shopping and resisting temptation (1.2, 4.1)
	Food labels	How to facilitate weight loss by understanding food labels (4.1)
	Portion sizes	Recommended portion sizes of different food groups to promote weight loss and maintenance (4.1)
	Healthy eating	Provides external links to healthy eating recipes to promote weight loss and maintenance (5.1, 9.1)
**Module 7: Physical activity, exercise, and NAFLD**		
	How lifestyle can contribute to weight gain and NAFLD	Animation communicating how reduced physical activity levels are liked to weight gain and NAFLD (5.2)
	Combining physical activity and diet to promote weight loss	The benefits of combining physical activity and dietary change to promote weight loss (5.1)
	Physical activity, exercise, and NAFLD	The difference between physical activity and exercise and how physical activity and exercise can benefit the liver and lead to other health benefits (5.1)
	How much physical activity do I need to do?	The required amount, intensity, and type of physical activity and exercise required to manage NAFLD and ideas for increasing physical activity and exercise levels (5.1)
**Module 8: Steps to success**		
	5 essential steps to goal setting	Tips for setting SMART^d^ goals (1.1)
	Behavioral goal setting	Tool to facilitate personalized behavioral goal setting for diet and physical activity (1.1)
	Dietary goal setting	Tool to facilitate monitoring of diet against dietary goals (2.3)
	Step tracker	Tool to facilitate monitoring of daily step counts and track progress over time (2.3)
	Weight tracking	Tool to facilitate weight monitoring and track progress (2.4)
	Maximizing chances of success	Animation prompting use of SMART goals and self-monitoring and support from family and friends to maximize the chances of success (1.1, 1.4, 2.3, 2.4, 3.1, 3.2)
Lifestyle coaching support		Review behavioral goals (1.5), provide feedback on behavior (2.2), and provide social support (unspecified; 3.1) and social support (practical; 3.2). Social support is directed specifically at the target behaviors

^a^BCT: behavior change technique.

^b^NAFLD: nonalcoholic fatty liver disease.

^c^NHS: National Health Service.

^d^SMART: Specific, Measurable, Achievable, Realistic, Timely.

### Intervention Mapping Step 5: Developing an Implementation Plan to Deliver VITALISE

Step 5 of the Intervention Mapping process involved working in collaboration with Changing Health Limited to develop an implementation plan. The plan consisted of training for lifestyle coaches, a standardized protocol for on-boarding (registration of patients) and making initial contact with patients, and allocation of coaching support.

A total of 3 lifestyle coaches employed by Changing Health Limited received training for the NAFLD prototype intervention. Training was delivered face-to-face in a single session by a senior physiotherapist with expertise in NAFLD and a health psychologist with expertise in lifestyle behavior change, and it lasted for 2 hours.

All 3 coaches were experienced in the delivery of lifestyle interventions in the context of long-term health conditions and providing remote support delivered by telephone. Therefore, the aims of the training session were to increase knowledge about NAFLD, including the management of NAFLD via lifestyle modification, and to further develop skills in delivering BCTs, specifically those incorporated within VITALISE (ie, strategies identified in step 3 to target motivation and enactment of behavioral change via engagement with the prototype digital intervention to promote weight loss and improve liver health).

The head of patient experience at Changing Health Limited agreed to make an initial contact with patients electing to take part in the program via email, providing them with a link to the digital intervention. If patients did not access the intervention within 7 days, a lifestyle coach assigned to VITALISE attempted to contact the patient via email and subsequently by telephone to prompt engagement. Changing Health agreed to provide patients with weekly lifestyle coaching sessions for 12 weeks during pilot testing of the intervention to allow them sufficient time and exposure to the intervention to provide feedback (step 6).

### Intervention Mapping Step 6: Pilot Testing the Intervention

With reference to the implementation plan referred to in step 5, 16 patients with NAFLD who agreed to participate in the pilot were given access to VITALISE for 12 consecutive weeks. It is important to record the way in which participants accessed the intervention as well as the feedback they provided to inform future development and evaluation. A total of 11 of the 16 participants successfully logged on to the digital intervention and entered details of their height, weight, and weight loss goal. Out of the 16 participants, 5 experienced problems when attempting to log in, and despite receiving a reset email and prompting from a lifestyle coach to access the intervention, they disengaged from it.

Table 4 presents a summary of the findings for the 11 participants who continued to engage with VITALISE beyond their first log-in. Each participant accessed the intervention an average of 4 times over the piloting period. They accessed the module content an average of 7 times per log-in. Goal setting and self-monitoring tools were accessed on 22 occasions, and the 11 participants accessed these tools 4 times each on average.

The modules accessed most frequently (between 7 and 10 unique accesses per participant) were *welcome to the program* (module 1), *understanding NAFLD* (module 2), and *food and NAFLD* (module 4). These modules were principally designed to target motivation for making lifestyle changes (behavioral intentions). The least frequently accessed modules covered topics to promote enactment of lifestyle behavior change, including goal setting, planning, problem solving, and monitoring. These findings supported the outcomes of the needs assessment conducted in step 1, where information about NAFLD was reported as most salient from the perspective of patients. Only 3 out of 11 participants requested access to a lifestyle coach for support throughout their use of the prototype intervention, and it was those participants who accessed the self-regulation tools (ie, goal setting and self-monitoring).

At the end of the 12-week piloting process, participants were emailed a brief qualitative questionnaire that asked them to provide written feedback on their experience of using the digital intervention. All 16 participants responded and provided feedback on the intervention, despite 5 being unable to access the intervention. When asked about the information content of the intervention, opinions varied between participants. For example, some reported the amount of information as too much, and at times difficult to understand, whereas others felt that the information provided was informative and pitched at the right level. The latter was particularly the case for participants who chose to access a lifestyle coach. A total of 2 participants indicated that they would usually prefer face-to-face support; however, they reported that the coaching support offered was innovative and an acceptable alternative. Those accessing a coach reported the support as beneficial for tailoring the information provided to their own individual circumstances. Participants reported that reviewing the information content motivated them to want to make lifestyle changes, but they did not provide any detail about why, for example, whether their primary motivator was liver health.

Access to VITALISE was reported to be the most salient barrier to engagement, and navigation for some was problematic, highlighting the need for further development, usability testing, and optimization. The participants who disengaged from the intervention did so due to several failed log-in attempts. As such, they reported losing confidence in the intervention despite being initially motivated to access it. It is important to note that the digital intervention was provided as a web link and not a mobile phone app. This was reported to affect initial motivation and engagement for those who expected to be able to access an app from the home screen of their mobile phone. This was reported to have reduced the number of times participants wanted to log-in (ie, ease of access would have promoted a higher level of use and engagement during the pilot). Those who disengaged and those who accessed the intervention reported this as having impacted their experience.

Access to the intervention was provided for 12 weeks; however, the majority of log-ins occurred within the first 4 weeks, where the information modules were accessed most frequently (ie, only 2 participants accessed the information module content on one occasion each after the initial 4 weeks, which corresponded to accessing a coach).

**Table 4:** Engagement with Intervention to Promote Lifestyle Change in Nonalcoholic Fatty Liver Disease by patients over a 12-week period.

Log-ins			Participants^a^	
**Distinct log-ins**				
	Total log-ins, n		57	
	**Log-ins per person**			
		Mean (SD)		3.6 (3.5)
		Range		0-14
		Median (IQR)		4 (5)
**Access to module content across 33 subsections**				
	Total views across all modules and subsections, n		208	
	**Views per section**			
		Mean (SD)		6.3 (0.7)
		Range		4-10
		Median (IQR)		7 (4)
**Access to goal setting and monitoring tools (5 individual tools available)**				
	Total log-ins, n		22	
	**Log-ins across all 5 tools**			
		Mean (SD)		4.4 (0.7)
		Range		3-6
		Median (IQR)		4 (2)

^a^This refers to 11 out of 16 (69%) participants originally recruited who logged in to the intervention.

## Discussion

### Principal Findings

The first-line treatment for NAFLD is lifestyle behavior change to promote weight loss and liver health [4,5]. Nevertheless, behavior change strategies and structured education for NAFLD are not routinely used in clinical care [9]. This paper describes the systematic development of a co-designed, theory- and evidence-informed digital intervention—VITALISE—designed to target dietary and physical activity behaviors of adults with NAFLD, to initiate and maintain weight loss to improve liver health. VITALISE addresses the pressing need for a structured lifestyle program for people with NAFLD with evidence-based information and behavior change strategies with support from a lifestyle coach to maximize engagement.

### Summary of Findings

Our qualitative study highlighted the need for information for patients about NAFLD, how it can progress, and how it can be managed. A minority of patients highlighted the need for support to make and sustain behavioral changes. Primary HCPs reported the need for information about NAFLD to raise their awareness and training to effectively communicate with patients about NAFLD and to provide support when required. Primary and secondary HCPs highlighted the importance of a referral pathway for patients to receive support to lose weight. They acknowledged a lack of skills in this area and a lack of time during consultations to effectively support patients to make behavioral changes [9]. Interview data were further analyzed using the TDF to identify targets for intervention. A total of 9 and 4 theoretical domains were identified in the context of HCP and patient interviews, respectively. These domains were used to select BCTs using a valid and reliable behavior change technique taxonomy [12].

### Relation to Other Literature in the Field

Intervention mapping enabled the development of an intervention that addressed the specific needs and preferences of patients and HCPs identified, with content informed by theory and practical theory-linked behavioral strategies to engage patients in a change process at their own pace and stage of readiness. This is consistent with previous research evidence on the capability of digital interventions to provide an effective medium for targeting social cognitive determinants of behavior [19], including diet and physical activity [20].

The opportunity for patients to engage with lifestyle coaches by telephone has been found to increase engagement with digital interventions [21,22] and to overcome barriers to goal attainment for diet and physical activity behavior change [22]. The inclusion of specifically trained coaches in the use of lifestyle interventions to manage NAFLD was viewed as an essential, but underutilized, component of VITALISE. A total of 3 out of 11 patients accessed coaching support during the piloting of the intervention and were very positive about the experience. This indicates that some patients may prefer to access this additional support immediately as they are ready to make changes. Our experience with development of other digital interventions has indicated that only around 40% to 50% of patients want to access a coach at the commencement or early stages of an intervention [22]. In the context of this study, it is possible that individuals accessed activities or support elsewhere or that they were not ready to make behavioral changes during the pilot phase. This needs to be explored further.

Modules 1, 2, and 4 were most often accessed, which could be explained by tunneling (delivery of the content in a prespecified order) when navigating the modules, although evidence has shown that the use of a free-roam (navigation in any order) did not improve engagement with or effectiveness of a digital brief alcohol intervention [27]. Therefore, it is possible that the tunneling of content and/or the amount of information provided by the digital intervention could have been overwhelming. Consequently, patients may not have persevered with the intervention long enough to gain access to a lifestyle coach. However, it is also possible that participants did not want to access a lifestyle coach and that information or education was sufficient for their needs. This assumption is congruent with the findings of our initial qualitative work [9], which was reported as part of step 1 of the development process.

### Limitations

Although our findings are encouraging, they also suggest that further development and optimization is required to maximize engagement with VITALISE. The need to summarize some of the information provided in VITALISE, and identifying what is critical to access before gaining access to a lifestyle coach is warranted in future work. Specifically, further exploration is required to determine why most participants did not access coaching support. The intervention was made available to participants for 12 consecutive weeks, and this included access to coaching support (ie, 1 session per week for 12 weeks), and we acknowledge that this is likely to be insufficient to promote long-term behavior change. However, the aim of the pilot study was to obtain data on access and use of the intervention to inform optimization and not to assess changes in behavior.

### Future Directions

The findings from this study have identified the need to further explore ways to maximize engagement with the intervention and utilization of coaching support. There is also a need to assess the feasibility of using the intervention within the clinical care pathway before assessing efficacy in a randomized controlled trial. A referral from an HCP may increase long-term engagement with the intervention when paired with positive reinforcement during annual review consultations [28], and this will be assessed. Future work will also involve the development and evaluation of a theory- and evidence-based training intervention for HCPs, which would accompany the patient intervention to support implementation at scale. On the basis of the findings from our qualitative work with HCPs, this is likely to include training for HCPs on how to provide information to patients on the importance of initiating lifestyle changes at the point of NAFLD diagnosis (to increase uptake of VITALISE), and personalized feedback for patients on the relationship between lifestyle behavior changes and clinical markers (eg, how changes in diet, physical activity, and weight loss impact blood test results [liver enzymes or glucose control] or specific liver investigations).

Although VITALISE was not piloted as part of clinical care delivery, it is easily embedded within the existing pathway of care. It provides HCPs in primary, secondary, and tertiary care centers with a mechanism to refer NAFLD patients for specialist behavior change support. This promotes more efficient use of clinicians’ time, enabling them to focus on their core clinical roles. However, patients considered it important for HCPs to acknowledge any progress they had made using the digital program during review appointments, specifically about the impact of behavioral changes on clinical outcomes. This suggests a need to include a summary report for patients to take along to clinical appointments with their HCPs, or with permission from patients, HCPs are given access to an electronic summary sent to patient medical records. Additional benefits of the digital intervention are that it can be tailored to individual patient needs and is capable of being delivered at scale. Furthermore, it incorporates NAFLD clinical management guidelines and has the potential to be implemented for use as part of the NAFLD care pathway in both primary and secondary care.

### Conclusions

In conclusion, intervention mapping provided a systematic user-centered method for developing a digital intervention with lifestyle coach support—VITALISE—for patients with NAFLD. The digital intervention was acceptable to patients and feasible to deliver. However, issues with initial access, optimization of information content, and promoting the value of remote support require further development ahead of future research to formally assess the acceptability and feasibility of the intervention in clinical settings and to establish the effectiveness of the intervention when delivered as part of existing care pathways.

## Abbreviations

BCT: behavior change technique
HAPA: health action process approach
HCP: health care professional
NAFLD: nonalcoholic fatty liver disease
NHS: National Health Service
TDF: Theoretical Domains Framework
VITALISE: Intervention to Promote Lifestyle Change in Nonalcoholic Fatty Liver Disease
